# Supplementary figures and images for: Using QRS loop descriptors to characterize the risk of sudden cardiac death in patients with structurally normal hearts
Source: PLoS One. 2022 Feb 16;17(2):e0263894. doi: 10.1371/journal.pone.0263894 (PMC8849494; doi:10.1371/journal.pone.0263894)

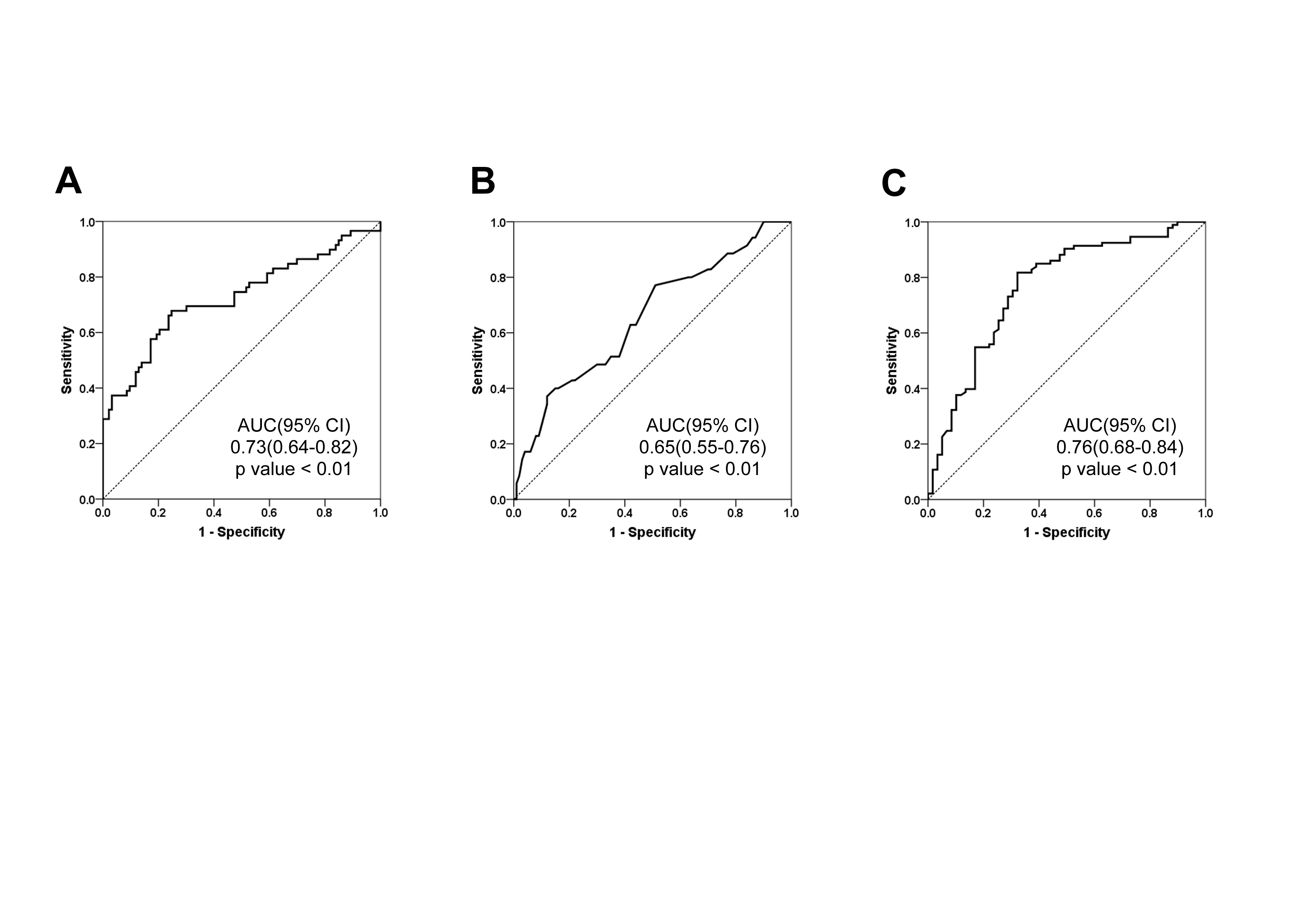

Supplement: S1 Fig — (A) The V4-5 dispersion value was a significant discriminant parameter in predicting sudden cardiac death(SCD), with an area under the curve(AUC) of 0.73 (95% confidence interval[CI], 0.64–0.82). (B) The QRS duration was a significant discriminant parameter in predicting SCD, with an AUC of 0.65(95% CI, 0.55–0.76). (C) The percentage of the loop area was a significant discriminant parameter in predicting non-SCD with an AUC of 0.76 (95% CI 0.68–0.84) (Abbreviation: AUC, area under the curve). (TIF) [file pone.0263894.s001.tif]

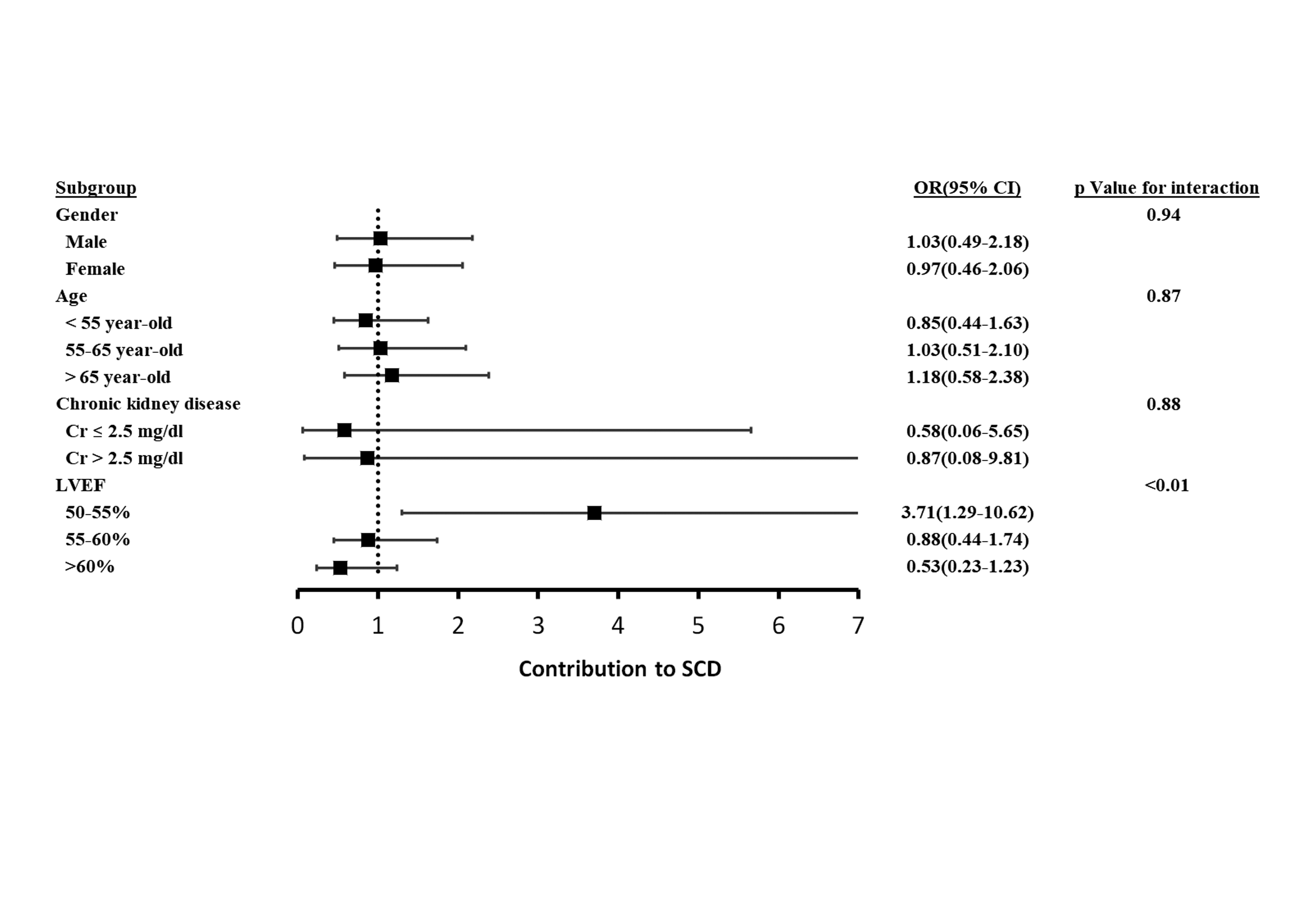

Supplement: S2 Fig — We performed a subgroup analysis to determine the risk factors in the two groups. We found that sex, age, and creatinine level did not significantly contribute to the incidence of SCD. Instead, we observed that the patients with a relatively low LVEF had a tendency to have a history of aborted SCD, although this was not significant. (Abbreviations: Cr, creatinine; LVEF, left ventricular ejection fraction; OR, odds ratio; QTc interval, corrected QT interval; SCD, sudden cardiac death). (TIF) [file pone.0263894.s002.tif]
