# Supplementary material for: Using QRS loop descriptors to characterize the risk of sudden cardiac death in patients with structurally normal hearts
Source: PLoS One. 2022 Feb 16;17(2):e0263894. doi: 10.1371/journal.pone.0263894 (PMC8849494; doi:10.1371/journal.pone.0263894)
Supplement: S1 Table — We used a cut-off value of >60° for V4-5 dispersion to characterize patients at risk for sudden cardiac death. (DOCX) [file pone.0263894.s003.docx]

| **S1 Table.** **Characteristics of patients with V4-5 dispersion > 60°** | |
| --- | --- |
| **Variables** | **N=15** |
| Age, mean ± standard deviation -yr | 51 ± 20.1 |
| Male- No.(%) | 11(73.3) |
| Underlying diseases |  |
| Hypertension- No.(%) | 5(33.3) |
| Old cerebrovascular accident- No.(%) | 1(6.7) |
| Diabetes mellitus- No.(%) | 1(6.7) |
| Chronic kidney disease- No.(%) | 0 |
| Hyperlipidemia- No.(%) | 1(6.7) |
| Chronic obstructive pulmonary disease- No.(%) | 2(13.3) |
| Coronary artery disease- No.(%) | 5(33.3) |
| Malignancy- No.(%) | 1(6.7) |
| Smoking- No.(%) | 1(6.7) |
| Etiology of sudden cardiac death |  |
| Idiopathic- No.(%) | 13(86.7) |
| Long QT syndrome- No.(%) | 0 |
| Brugada syndrome- No.(%) | 1(6.7) |
| Arrhythmogenic right ventricular cardiomyopathy- No.(%) | 1(6.7) |
| Echocardiogram parameters |  |
| LVEF-% | 56.7 ± 4.1 |
| IVS- mm | 8.6 ± 1.0 |
| LVIDED- mm | 48.4 ± 5.1 |
| ECG parameters |  |
| Heart rate- beats/min | 74.4 ± 21.2 |
| PR interval- ms | 175.5 ± 19.0 |
| QRS duration- ms | 100.5 ± 7.0 |
| QTc- ms | 463.3 ± 53.1 |
| LVH- No.(%) | 0 |
| Bundle branch block- No.(%) | 0 |
| Pathological Q wave- No.(%) | 0 |
| TWI ≥ V2- No.(%) | 0 |
| Vectorcardiographic parameters |  |
| V_1-2_ dispersion- ° | 50.8 ± 17.8 |
| V_2-3_ dispersion- ° | 51.6 ± 18.0 |
| V_3-4_ dispersion- ° | 41.1 ± 19.3 |
| V_4-5_ dispersion- ° | 69.6 ± 6.5 |
| V_5-6_ dispersion- ° | 29.8 ± 19.7 |
| V_6_-I dispersion- ° | 65.3 ± 14.4 |
| Loop dispersion- N | 306.9 ± 31.4 |
| Percentage of loop area- % | 57.7 ± 9.5 |
| ECG, electrocardiography. IVS, interventricular septum. LVIDED, left ventricular inner dimension at end diastole. LVEF, left ventricular ejection fraction. LVH, left ventricular hypertrophy by Sokolow–Lyon index >35 mm. QTc, corrected QT interval. TWI ≥ V2, T wave inversion beyond V1. | |
