# Supplementary material for: Using QRS loop descriptors to characterize the risk of sudden cardiac death in patients with structurally normal hearts
Source: PLoS One. 2022 Feb 16;17(2):e0263894. doi: 10.1371/journal.pone.0263894 (PMC8849494; doi:10.1371/journal.pone.0263894)
Supplement: S2 Table — Receiver operating characteristic curve analysis was used to assess the values of the continuous variables for risk stratification, and optimal cutoff values were calculated sequentially according to the specificity and sensitivity. (DOCX) [file pone.0263894.s004.docx]

| **S2 Table.** **ROC analysis of parameters for predicting SCD or non-SCD** | | | | | | |
| --- | --- | --- | --- | --- | --- | --- |
| **Parameters** | **Area under the ROC curve (95% CI)** | **Cutoff point** | **Specificity(%)** | **Sensitivity(%)** | **PPV(%)** | **NPV(%)** |
| **For predicting SCD** | | | | | | |
| **V_4-5_ dispersion- °** | 0.73 (0.64-0.82) | 37.7 | 75.3 | 67.8 | 62.5 | 78.4 |
| **QRS duration- ms** | 0.65 (0.55-0.76) | 89.0 | 49.5 | 77.1 | 34.6 | 86.0 |
| **For predicting non-SCD** | | | | | | |
| **Percentage of loop area- %** | 0.76 (0.68-0.84) | 62.6 | 67.8 | 81.7 | 80.0 | 70.2 |
| CI, confidence interval. NPV, negative predictive value. PPV, positive predictive value. ROC, receiver operating characteristic curve. SCD, sudden cardiac death. | | | | | | |
