# Supplementary material for: Using QRS loop descriptors to characterize the risk of sudden cardiac death in patients with structurally normal hearts
Source: PLoS One. 2022 Feb 16;17(2):e0263894. doi: 10.1371/journal.pone.0263894 (PMC8849494; doi:10.1371/journal.pone.0263894)
Supplement: S3 Table — There was no difference in the underlying diseases, vectorcardiographic parameters, and most ECG and echocardiographic parameters between the two groups. Only the PR interval was significantly longer in the shockable rhythm group than in the other group. (DOCX) [file pone.0263894.s005.docx]

| **S3 Table. Baseline characteristics of the SCD patients with shockable and non-shockable rhythm** | | | | |
| --- | --- | --- | --- | --- |
|  | **Total**  **(N=59)** | **Shockable rhythm (N=25)** | **Non-shockable rhythm**  **(N=34)** | ***p* value^a^** |
| **Age-yr** | 55.3 ± 17.5 | 53.0 ± 19.4 | 57.0 ± 16.1 | 0.39 |
| **Male- No. (%)** | 45(76.3) | 19(76.0) | 26(76.5) | 0.60 |
| **Hypertension- No. (%)** | 23(39.0) | 12(48.0) | 11(32.4) | 0.17 |
| **Old CVA- No. (%)** | 1(1.7) | 1(4.0) | 0(0) | 0.42 |
| **Diabetes mellitus- No. (%)** | 4(6.8) | 1(4.0) | 3(8.8) | 0.43 |
| **Chronic kidney disease- No. (%)** | 2(3.4) | 0(0) | 2(5.9) | 0.33 |
| **Hyperlipidemia- No. (%)** | 7(11.9) | 4(16.0) | 3(8.8) | 0.33 |
| **COPD- No. (%)** | 2(3.4) | 2(8.0) | 0(0) | 0.18 |
| **Coronary artery disease- No. (%)** | 11(18.6) | 5(20.0) | 6(17.6) | 0.54 |
| **Malignancy- No. (%)** | 1(1.7) | 1(4.0) | 0(0) | 0.42 |
| **Smoking- No. (%)** | 1(1.7) | 1(4.0) | 0(0) | 0.42 |
| **Echocardiogram** |  |  |  |  |
| **LVEF-%** | 58.0 ± 4.8 | 58.7 ± 4.9 | 56.8 ± 4.6 | 0.23 |
| **IVS- mm** | 8.4 ± 1.0 | 8.6 ± 0.9 | 8.2 ± 0.9 | 0.08 |
| **LVIDED- mm** | 48.2 ± 4.5 | 47.9 ± 5.2 | 48.4 ± 4.0 | 0.63 |
| **ECG parameters** |  |  |  |  |
| **Heart rate- beats/min** | 72.0 ± 16.2 | 68.3 ± 13.6 | 74.8 ± 17.7 | 0.14 |
| **PR interval- ms** | 169.1 ± 22.7 | 179.4 ± 14.2 | 164.0 ± 20.1 | 0.01 |
| **QRS duration- ms** | 95.7 ± 10.3 | 96.8 ± 10.8 | 94.9 ± 10.1 | 0.60 |
| **QTc- ms** | 456.0 ± 6.5 | 464.8 ± 48.5 | 448.8 ± 48.7 | 0.23 |
| **LVH- No.(%)** | 0 | 0 | 0 | - |
| **BBB- No.(%)** | 0 | 0 | 0 | - |
| **Pathological Q wave- No.(%)** | 0 | 0 | 0 | - |
| **TWI ≥ V2- No.(%)** | 2(1.9) | 2 | 0 | 0.18 |
| **Vectorcardiographic parameters** |  |  |  |  |
| **V_1-2_ dispersion- °** | 48.7 ± 20.3 | 51.0 ± 17.9 | 46.9 ± 21.9 | 0.45 |
| **V_2-3_ dispersion- °** | 52.0 ± 19.5 | 46.3 ± 17.0 | 56.2 ± 20.4 | 0.05 |
| **V_3-4_ dispersion- °** | 44.5 ± 18.3 | 42.7 ± 16.8 | 45.8 ± 19.4 | 0.51 |
| **V_4-5_ dispersion- °** | 44.0 ± 20.2 | 46.7 ± 18.1 | 42.1 ± 21.7 | 0.39 |
| **V_5-6_ dispersion- °** | 28.8 ± 18.3 | 25.6 ± 13.4 | 31.1 ± 21.1 | 0.26 |
| **V_6_-I dispersion- °** | 66.9 ± 13.9 | 66.9 ± 12.9 | 67.0 ± 14.9 | 0.98 |
| **Loop dispersion- N** | 301.6 ± 28.0 | 305.6 ± 36.5 | 298.5 ± 19.8 | 0.34 |
| **Percentage of loop area- %** | 55.0 ± 14.0 | 54.7 ± 14.3 | 55.9 ± 14.0 | 0.75 |
| BBB, bundle branch block. COPD, chronic obstructive pulmonary disease. CVA, cerebrovascular accident. ECG, electrocardiography. IVS, interventricular septum. LVIDED, left ventricular inner dimension at end diastole. LVEF, left ventricular ejection fraction. LVH, left ventricular hypertrophy by Sokolow–Lyon index >35 mm. QTc, corrected QT interval. SCD, sudden cardia death. TWI ≥ V2, T wave inversion beyond V1.  ^a^P values were between SCD patients with and without shockable rhythm. | | | | |
