# Supplementary material for: Using QRS loop descriptors to characterize the risk of sudden cardiac death in patients with structurally normal hearts
Source: PLoS One. 2022 Feb 16;17(2):e0263894. doi: 10.1371/journal.pone.0263894 (PMC8849494; doi:10.1371/journal.pone.0263894)
Supplement: Attachment — Submitted filename: Response_to_Reviewers_R1.docx [file pone.0263894.s006.docx]

We thank the academic editor and reviewers for the very useful comments. Those comments were very instructive and very helpful to this manuscript. You will find our response to your comments below (in blue color).

**Response to Journal Requirements**

We would like to thank you for evaluating our manuscript and reminding us the valuable comments. Following your suggestion, we have modified manuscript in accordance with Journal Requirements.

1. Regarding the journal requirements: “please ensure that your manuscript meets PLOS ONE's style requirements.”

Thank you for this reminder very much. Following the guidance, we have ensured the manuscript meets PLOS ONE's style requirements accordingly.

2. Regarding the journal requirement: “please provide additional details regarding participant consent. In the ethics statement in the Methods and online submission information, please ensure that you have specified (1) whether consent was informed and (2) what type you obtained (for instance, written or verbal, and if verbal, how it was documented and witnessed). If your study included minors, state whether you obtained consent from parents or guardians. If the need for consent was waived by the ethics committee, please include this information. If you are reporting a retrospective study of medical records or archived samples, please ensure that you have discussed whether all data were fully anonymized before you accessed them and/or whether the IRB or ethics committee waived the requirement for informed consent. If patients provided informed written consent to have data from their medical records used in research, please include this information.”

Thank you for these reminders very much. Following your guidance, we have ensured the additional details regarding participant consent has been provided in the section of Materials and methods in lines 78-81 on page 4 and online submission information.

3. Regarding the comments: “we suggest you thoroughly copyedit your manuscript for language usage, spelling, and grammar. If you do not know anyone who can help you do this, you may wish to consider employing a professional scientific editing service.”

Thank you for your comments very much. Following your recommendation, we considered employing a professional scientific editing service, and we have provided a copy of the manuscript as a *supporting_information* file showing the changes by professional scientific editing service.

4. Regarding the comments: “we note that you have provided funding information that is not currently declared in your Funding Statement. However, funding information should not appear in the Acknowledgments section or other areas of your manuscript. We will only publish funding information present in the Funding Statement section of the online submission form. Please remove any funding-related text from the manuscript and let us know how you would like to update your Funding Statement. Please include your amended statements within your cover letter; we will change the online submission form on your behalf.

Thank you for your comments very much. We have modified the section of Acknowledgments in line 420 on page 20 as follows: “none.” In addition, the amended Funding Statements have been provided in the cover letter, and thank you for changing the online submission form on my behalf.

5. Regarding the comments: “we note that the grant information you provided in the ‘Funding Information’ and ‘Financial Disclosure’ sections do not match. When you resubmit, please ensure that you provide the correct grant numbers for the awards you received for your study in the ‘Funding Information’ section.”

Thank you for your comments very much. We have ensured the correct grant numbers for the awards being provided in Funding Information and Financial Disclosure.

**Response to Reviewer #1**

We want to thank you for evaluating our manuscript and providing us with valuable comments. Following your suggestion, we have modified several parts of our manuscript accordingly.

1. Regarding the comment: "Were only patients with a presumed cardiac cause of SCD included, and e.g. patients with cardiac arrest due to pulmonary embolism or other non-cardiac causes excluded? I cannot find this in the in- or exclusion criteria."

Thank you for this comment very much. We only included the patients with a presumed cardiac cause of SCD because we aimed to identify the abnormal substrates of the ventricle that are difficult to access by electrocardiography and echocardiography to define high-risk patients vulnerable to SCD. Following your comment, we have modified the section of Methods by adding a sentence in line 88 on page 4 as follows: "patients with a history of aborted SCD due to non-cardiac causes were also excluded."

2. Regarding the comments: "The etiology of ventricular arrhythmic SCD and non-arrhythmic SCD are often very different. Why have the authors decided to lump together ventricular arrhythmia and non-VA SCD? This is my main concern about this study. I would at least prefer to see a subgroup analysis of the studied parameters between VA and non-VA SCD. Were there also patients who presented with non-shockable rhythms initially but who had shockable rhythms later on during resuscitation? Also, the proportion of ventricular arrhythmia of these patients who have survived SCA is quite low, which is surprising considering the percentage of initial presentation VA is similar to the percentage provided here: ~37% (e.g. Oving et al. Occurrence of shockable rhythm in out-of-hospital cardiac arrest over time: A report from the COSTA group. Resuscitation 2020 <https://www.sciencedirect.com/science/article/pii/S0300957220301258>), but also the usually much higher rate of survival among SCA patients who present with VA. (e.g. Berdowski et al. Global incidences of out-of-hospital cardiac arrest and survival rates: Systematic review of 67 prospective studies. Resuscitation. 2010 <https://www.sciencedirect.com/science/article/abs/pii/S0300957210004326>) The authors later say the real incidence of VA in SCD patients remained unknown because of inevitably progresses to unwitnessed asystole', however, this does not explain the large difference between this study and others. Can the authors provide details on why this relatively low percentage of VA/shockable rhythm survivors (or high percentage of non-shockable rhyhm survivors) has occurred?"

Thank you for this comment very much. Regarding the first question, the method we used to lump these 2 types of SCD together was based on the previous literature (Huikuri, et al. N Engl J Med 2001) in which the majority of SCD was proposed to result from malignant arrhythmias, although VAs were not frequently detected. VA and non-VA were considered to be in the same pathophysiology cascade but at different time points. Malignant arrhythmias probably have already degenerated to asystole, or sinus rhythm has already been restored when there is no detectable VA in aborted SCD. Therefore, we aimed to identify these abnormal substrates of the ventricle that are difficult to access by electrocardiography and echocardiography to define high-risk patients vulnerable to sudden cardiac death. In order to clarify this point of view, we have added the statements into the section of introduction in lines 61-64 on page 3 as follows: "VAs and non-VAs were considered to be in the same pathophysiology cascade but occur at different time points. Malignant arrhythmias probably have already degenerated to asystole, or sinus rhythm has already been restored when there is no detectable VA in aborted SCD."

Following your comment, we also added a subgroup analysis in the S3 Table to compare the studied parameters between patients with and without initial VAs(or shockable rhythms). There was no difference in the underlying diseases, vectorcardiographic parameters, and most ECG and echocardiographic parameters between the two groups. Only the PR interval was significantly longer in the shockable rhythm group than in the other group, which was probably attributed to the effect of multiple factors, including the heart rate. Given these results, we also added the statements in the section of Discussion in lines 303-308 on page 16 as follows: "in addition, the baseline demographic characteristics and studied parameters were also compared between the patients with and without shockable rhythms at initial presentation (S3 Table). There was no difference in the underlying diseases, vectorcardiographic parameters, and most ECG and echocardiographic parameters between the two groups. Only the PR interval was significantly longer in the shockable rhythm group than in the other group, which was probably attributed to the effect of multiple factors, including the heart rate."

As to the second question, the shockable rhythm in our study was defined as showing in the initial rhythm or during the resuscitation. Therefore, it is difficult to determine if patients presented non-shockable rhythms initially and had shockable rhythms later on during resuscitation. We have added the statements in the section of Methods to clarify this part in lines 93-94 on page 4 as follows: "a shockable rhythm was presented as the initial rhythm or during resuscitation."

Regarding the third question and the last part in this comment, we agreed with you that the proportion of VA (42.4%) among the survivors in this study was relatively low. According to the pooled dataset from the COSTA group, the percentage of patients presenting with VA among European patients who experienced out-of-hospital cardiac arrest (OHCA) has already reached 37–38%. However, the differences between continents and ethnicities in these data probably need to be considered. A lower detection rate of a shockable rhythm at the initial presentation in Asia has been reported by Berdowski et al (Berdowski, et al. Resuscitation 2010). In addition, real-world data from the THUNDER study (Lin, et al. Mayo Clin Proc 2017) based on the Taiwanese population also disclosed that the incidence of a shockable rhythm in patients who experienced OHCA was only 7.9%. According to this registry, 17.8% of the patients with an initial shockable rhythm and 2.7% of those with an initial non-shockable rhythm would be able to survive to discharge, indicating that only 36% of all survivors who experienced OHCA had shockable rhythms at the initial presentation. Taken together, our results are similar to the real-world Taiwanese registry data and was consistent with other study data in relation to the difference among continents. In order to elucidate this viewpoint, we have added the statements in the section of Discussion in lines 377-390 on page 19 as follows: "the proportion of VA (42.4%) among the survivors in this study was relatively low. According to the pooled dataset from the COSTA group, the percentage of patients presenting with VA among European patients who experienced out-of-hospital cardiac arrest (OHCA) has already reached 37–38%. However, the differences between continents and ethnicities in these data probably need to be considered. A lower detection rate of a shockable rhythm at the initial presentation in Asia has been reported by Berdowski et al. In addition, real-world data from the THUNDER study based on the Taiwanese population also disclosed that the incidence of a shockable rhythm in patients who experienced OHCA was only 7.9%. According to this registry, 17.8% of the patients with an initial shockable rhythm and 2.7% of those with an initial non-shockable rhythm would be able to survive to discharge, indicating that only 36% of all survivors who experienced OHCA had shockable rhythms at the initial presentation. Taken together, our results are similar to the real-world Taiwanese registry data and was consistent with other study data in relation to the difference among continents."

3. Regarding the comment: "could the authors elaborate a bit more on the survivorship bias(Discussion section, page 17, line 331)? There is a possibility that these findings relate to the survival of cardiac arrest and not to the risk of cardiac arrest itself. Also, because these ECG's were collected after the event, the differences found may also be a result of the event itself and, for example, its associated cardiac ischaemia instead of a marker of sudden cardiac death. Overall, I think the conclusion that “low PL and high V4-5 QRS dispersion values were associated with increased SCD risks in patients with structurally normal hearts.” (Conclusion section, page 17, line 342-344) is too strong and should be rephrased into something like “were associated with patients who survived SCD”, since, based on this data we cannot say for certain it is also found in people who are at risk of SCD, but in whom SCD hasn’t occurred yet."

Thank you for this comment very much. We agree with you that survivorship bias is probably associated with these findings of survival of cardiac arrest. It was also one of the limitations in our study because we had no assessment of those patients who didn't survive from OHCA. This comment is very precious, and we have discussed this in the section of Limitations in lines 392-399 on pages 19-20 as follows: "there is a possibility of selection bias and survivorship bias because only patients with a cerebral performance category score equal to 1 were enrolled. We had no access to the patients who didn't survive the OHCA as well as the severely ill patients who were unable to undergo 1-min vectorcardiography owing to poor clinical status; therefore, the findings of our study were probably related to the survival of cardiac arrest instead of the risk of the cardiac arrest itself. In addition, the ECG parameters were collected and elaborated after the event; thus, the differences found in our study might also have a chance to be a result of these events." We also agree with your valuable comment on the conclusion, and we have rephrased the statements in lines 416-417 on page 20 as follows: "low PL and high V_4-5_ QRS dispersion values are associated with patients who had structurally normal hearts and experienced aborted SCD."

4. Regarding the comment: "Is there a more contemporary reference than this one from 1998?"

Thank you for this comment very much. Following your comment, we have modified this statement and added more contemporary references in line 51 and page 3.

5. Regarding the comment: "Some words like "a", "the" and "are" are left out, or added unnecessarily. Although small, these words are important for the sentence structure and would improve the readability of the manuscript. For example page 3, line 57-62. “In [a] healthy population without structural heart disease screened by echocardiography or ECG, some anomalies might exist but [are] hard to be identified, especially [[the (can be left out)]] electrical abnormalities susceptible to malignant arrhythmias. Although ventricular arrhythmias (VA) were not frequently detected, [a] previous study has proposed that the majority of SCD could probably result from malignant arrhythmias.” In figure S2, the authors have written "chornic" instead of "chronic" kidney disease.

Thank you for this comment very much. We have modified these words in lines 57-61 on page 3 and S2 Fig accordingly.

**Response to Reviewer #2**

We want to thank you for evaluating our manuscript and providing us with valuable comments. Following your comments, we have modified several parts of our manuscript accordingly.

1. Regarding the comment: "the population studied (both cases and controls) appears to be highly selected. One would expect to see a higher proportion of subjects with aborted sudden cardiac death to have coronary artery disease, and a smaller proportion to be idiopathic. It would be of use to describe in more detail how the subjects were included (what was the indication to record vectorcardiography?). Then the reader could better think about the generalizability of the findings. Were the controls healthy volunteers, patients of an arrhythmia clinic without aborted sudden cardiac death, patients with syncope, or something else? How big proportion of patients with aborted sudden cardiac death in the area during the study period ended up in the study? Also, to assess the value of the of the electrocardiographic variables studied in risk stratification it would be of use to know the distribution of these variables in healthy subjects."

Thank you for these comments very much, and these questions are very important to help readers think about the generalizability of our findings. As to the first and second questions, our cases were enrolled to undergo vectorcardiography from the clinics during the study period, and all these patients had histories of either aborted SCD or palpitation. Following your comments, we have modified the statements in lines 84-86 on page 4 as follows: "this study consecutively enrolled patients who had histories of aborted SCD or palpitation in our clinics and undergo vectorcardiography between March 2017 and December 2018."

As to the third question, unfortunately, we didn't have a detailed number of aborted SCD in this area during the study period. However, an estimation can be made according to a previous study that was conducted in central Taiwan with a population of 2.7 million (Lin, et al. Mayo Clin Proc 2017). In that study, there were 1629 OHCA patients in a year, and 3.9% of these patients survived. Our hospital is a tertiary referral medical center in the Taipei metropolitan area that encompasses a population of 6.6 million. Hence, at a rough estimate, there were 285 patients with aborted SCD in this region during the study period, and 25.3% of these patients were referred to our hospital. Following your comment, we have added the statements in the section of Limitations in lines 401-408 on pages 20 as follows: "our hospital is a tertiary referral medical center in the Taipei metropolitan area, which encompasses a population of 6.6 million. Unfortunately, we did not have a detailed number of aborted SCD events in this area; thus, we could not precisely assess the proportion of patients included in this study. However, an estimation can be made according to a previous study conducted in central Taiwan with a population of 2.7 million. In that study, there were 1629 patients who experienced OHCA in a year, of whom 3.9% survived. Hence, at a rough estimate, there were 285 patients with a history of aborted SCD in this region during the study period, of whom 25.3% were referred to our hospital."

Following the last comment, we added S1 Fig and S2 Table to demonstrate using ROC curve analysis to elaborate the values of these parameters for risk stratification. We have added the statements in the section of Method in lines 167-169 on page 7 as follows: "receiver operating characteristic (ROC) curve analysis was used to assess the values of the continuous variables for risk stratification, and optimal cutoff values were calculated sequentially according to the specificity and sensitivity." We have also added descriptions for S1 Fig and S2 Table in the section of Results in line 259-268 on pages 13-14 as follows: "moreover, we used ROC curve analysis to assess the values of the PL, V_4-5_ dispersion, and QRS duration for predicting SCD or non-SCD (S1 Fig), and the summary is shown in S2 Table. For predicting SCD, the area under the curve (AUC) for the V_4-5_ dispersion value was 0.73 (95% CI, 0.64–0.82, S1A Fig) with an optimal cut-off value of 37.7° (specificity, 75.3%; sensitivity, 67.8%; positive predictive value (PPV), 62.5%; negative predictive value (NPV), 78.4%); that for the QRS duration was 0.65(95% CI, 0.55–0.76, S1B Fig), with an optimal cut-off value of 89 ms (specificity, 49.5%; sensitivity, 77.1%; PPV, 34.6%; NPV, 86.0%). For predicting non-SCD, the AUC for the PL was 0.76 (95% CI, 0.68–0.84, S1C Fig), with an optimal cut-off value of 62.6% (specificity, 67.8%; sensitivity, 81.7%; PPV, 80.0%; NPV, 70.2%)."

2. Regarding the comment: "before proposing new risk markers one should assess whether the new markers provide new information. Two of the three example electrocardiograms are clearly abnormal. Please report the prevalence of electrocardiographic LVH, bundle branch blocks, T wave inversions, and Q waves in both cases and controls. Also please provide detailed echocardiographic data in addition to left ventricular ejection fraction (at least wall thickness and LVEDD)."

Thank you for this comment very much. Following your comment, we have added the variables, including electrocardiographic LVH by Sokolow–Lyon index >35 mm, bundle branch block, T wave inversion beyond V1, pathologic Q waves, thickness of interventricular septum, and left ventricular inner dimension at end-diastole, in Table 2 and S3 Table.

3. Regarding the comment: "there are "empty" dots in the box plots (Figure 3.). Is there some kind of a problem with the data?"

Thank you for this comment very much. There are no problems with this data, and the empty dots in these box plots represent the location of outliers. In these box plots, the distribution of the data is disclosed by the black dots, and the empty dots exhibit the location of outliers and line centrally. According to the statistical definition, a data dot is said to be an outlier if it is greater than the third quartile of data plus 1.5 times the interquartile range (high outlier) or less than the first quartile of data minus 1.5 times the interquartile range (lower outlier). For instance, in the left panel of Fig 3B, there are 8 black dots that are outliers in this graph, so the empty dots are repotted to disclose the location of these outliers and line centrally. Therefore, upper lines represent the maximum value in the dataset without high outliers, and lower lines represent the minimum value in the dataset without lower outliers. Follow this comment, I have added the statements in the figure legend of Fig 3 to make it clear in lines 226-233 on page 12 as follows: "boxes in the box plots start in the first quartile, end in the third quartile, and represent 50% of the central data. A line inside represents the median values. In these box plots, black dots present the distribution of the data, and empty dots exhibit the location of the outliers and line centrally. A data dot is said to be an outlier if it is greater than the third quartile of data plus 1.5 times the interquartile range (high outlier) or less than the first quartile of data minus 1.5 times the interquartile range (lower outlier). Therefore, upper lines represent the maximum value in the dataset without high outliers, and lower lines represent the minimum value in the dataset without lower outliers."

4. Regarding the comment: "the most interesting part of the population is the subset with V4-V5 dispersion >60 as one can see in Figure 3. that such high dispersion was not present in any of the controls. Thus, the use of this (or higher) cutoff might prove useful in identifying subjects at risk of sudden cardiac death. Please provide the charactestics, other electrocardiographic findings, ECHO findings, and identified channelopaties and other diseases of this group also."

Thank you for this comment very much. Following your recommendation, we use a cut-off of V_4-5_ dispersion > 60**°** to characterize the patients at risk of SCD, and we have provided the characteristics, and electrocardiographic and echocardiogram findings in S1 Table. We have also added the statements in the section of Results in lines 244-252 on page 13 as follows: "based on the findings shown in Fig 3A, there was no subject in the control group with a V_4-5_ dispersion value of >60°. To shed some light on the features of patients at risk for SCD, we demonstrated the baseline characteristics and ECG and echocardiographic findings of the patients with V_4-5_ dispersion values of >60° in S1 Table. We noticed that most of these patients were men (73.3%), and the mean age was 51 years. Histories of hypertension and coronary artery disease accounted for 33.3% of all cases. Regarding the etiologies of SCD, one case was Brugada syndrome (6.7%); one was ARVC (6.7%); and 13 were idiopathic (86.7%). In addition, there were no significant abnormalities in the ECG and echocardiographic parameters."

5. Regarding the comment: "the construction of the propensity score is not reported in detail. Please see for example Althouse AD et al. Recommendations for Statistical Reporting in Cardiovascular Medicine: A Special Report From the American Heart Association. Circulation 2021 for recommendation about reporting."

Thank you for this comment very much. We have modified the description accordingly in lines 94-96 on pages 4 as follows: " propensity score matching was performed to minimize the confounders. The cases and controls were matched at a 1:2 ratio using a 0.10 caliper for identical characteristics of age, sex, and histories of hypertension and coronary artery disease."

6. Regarding the comment: "the use of an expert scientific English editor would be beneficial to improve the readability of the manuscript."

Thank you for this comment very much. We have employed a professional scientific editing service to copyedit our manuscript for language usage, spelling, and grammar accordingly. A copy of the manuscript was provided as a *supporting_information* file showing the changes by professional scientific editing service.
