# Supplementary material for: Using QRS loop descriptors to characterize the risk of sudden cardiac death in patients with structurally normal hearts
Source: PLoS One. 2022 Feb 16;17(2):e0263894. doi: 10.1371/journal.pone.0263894 (PMC8849494; doi:10.1371/journal.pone.0263894)
Supplement: Attachment — Submitted filename: response_to_reviewers_R2.docx [file pone.0263894.s007.docx]

We thank the reviewer for the very useful comments. Those comments were very instructive and very helpful to this manuscript. You will find our response to your comments below (in blue color).

Response to Reviewer #2

Thank you for evaluating our manuscript and providing us with valuable comments. Following your comments, we have modified several parts of our manuscript accordingly.

1. Regarding the comment: “The Title of the study should be reconsidered as vectorcardiographic loop assessment and QRS duration were also among the main findings.”

Thank you for this comment very much. Following your comment, we have revised the title of the article as follows: “Using QRS loop descriptors to characterize the risk of sudden cardiac death in patients with structurally normal hearts.”

2. Regarding the comment: “The exact definition of structurally normal heart/structural heart disease should be provided. Which conditions/findings led to exclusion?”

Thank you for this comment very much. Following your comment, we have revised the section of Methods in lines 86-90 on page 4 as follows: “these patients didn't have structural heart diseases(e.g., dyskinesia or hypertrophy of left ventricles), histories of congestive heart failure, or reduced ejection fraction (<50%), and the attribution to non-cardiac causes wasn't favored in patients with a history of aborted SCD. A total of 315 patients were investigated.”

3. Regarding the comments: “The authors state that the control subjects indication for vectorcardiography was palpitationsWere the patients admitted to vectorcardiogram for clinical reasons, or for the purposes of the present study? Thorough characterization of the control subjects is of great importance to enable accurate interpretation of the results, and their generalizability. In the flow chart it should be reported that how many subjects underwent vectorcardiography during the time period, and how many of those subjects were excluded due to structural heart disease/reduced ejection fraction, how many due to lacking echocardiographic/other data, how many did not give concent. Also, it would be of great importance to know about the control subjects in more detail (What were the diagnoses after examinations? VES? Atrial ectopy? NSVT? Were subjects with syncope included?).”

Thank you for these comments very much. These control subjects were diagnosed having atrioventricular node reentrant tachycardia after examination and were admitted for therapy, and the vectorcardiography was also applied when the patients agreed with it. Following your comment, we have revised the statements in lines 84-86 on page 5 as follows: “this study consecutively enrolled patients who had histories of aborted SCD or palpitation that is due to atrioventricular node reentrant tachycardia in our clinics and underwent vectorcardiography between March 2017 and December 2018.”

Regarding the comment on the flow chart, because we only focused on the patients who had histories of aborted SCD or palpitation that is due to atrioventricular node reentrant tachycardia in our clinics and underwent vectorcardiography between March 2017 and December 2018, it would be difficult to conclude the actual numbers of other situations. In order to clarify the concept of enrollment, we have removed the terms(e.g., exclusion and inclusion) to make it clear and modified it in the section of Abstract(lines 30-33 on page 2) and Methodology(lines 86-90 on page 4) as follows: “these patients didn't have structural heart diseases, histories of congestive heart failure, or reduced ejection fraction, and they were classified into SCD (with aborted SCD history and cerebral performance category score of 1) and control groups (without SCD history),” and “these patients didn't have structural heart diseases(e.g., dyskinesia or hypertrophy of left ventricles), histories of congestive heart failure, or reduced ejection fraction (<50%), and the attribution to non-cardiac causes wasn't favored in patients with a history of aborted SCD. A total of 315 patients were investigated.”

4. Regarding the comment: “The authors should report how the vectorcardiograms were recorted. Was Dower transform used or did they use specific Frank electrode placements?”

We thank the reviewer for the suggestion. Instead of using a spatial filter with fixed coefficients to construct three orthogonal leads (i.e., Frank's leads X, Y, and Z) from 12 leads ECG, we studied the spatial variation in the space defined by the singular vectors; all QRS loop descriptors (PL, LD, IQRSD) are measured using the ECG vector in the constructed space spanned by the eigenvector of the ECG matrix.

We also provide how to derive a 3D representation of the cardiac electrical activity from a singular vector in sections 2.3 and 2.4. However, to avoid misunderstanding, the title of section 2.2. was replaced as “Morphological complexity of the 12-Lead QRS wave”. We also made some modifications in section 2.2 in lines 110-114 on page 5 as follows: "the 12-Lead ECG was taken at a stable stage using LabSystem^TM^ PRO (Boston Scientific, Boston, MA, USA) with a 1-min duration for QRS descriptor analysis. The QRS descriptors, including the percentage of the loop area (PL), loop dispersion (LD), and inter-lead QRS dispersion (IQRSD) are measured using the ECG vector in the constructed space spanned by the eigenvector of 12 lead ECG matrix.”

5. Regarding the comment: “Please state in the methods the definitions for hyperlipidemia, CKD, old CVA, and how the prevalence coronary artery disease was ascertained. Did the SCD cases undergo routine coronary angiography or CTA? What were the findings?”

Thank you for this comment very much. These targeted co-morbidities, including hyperlipidemia, CKD, old CVA, prior CAD, were determined by using the *International Classification of Diseases* (ICD) 9 codes from the medical record at the time of examination. Following your comment, we have stated it in the section of Methods in lines 117-119 on page 5 as follows: “Targeted co-morbidities, including hyperlipidemia, CKD, old CVA, prior CAD, were determined by using the *International Classification of Diseases* (ICD) 9 codes from the medical record at the time of examination.”

We agree with you that routine coronary angiography and CTA following SCD are required. In our study, all the patients with a history of aborted SCD have received routine coronary angiography, and none of them have acute coronary syndrome. We have added this statement in the section of Results in lines 253-254 on page 13 as follows: “all the patients with a history of aborted SCD have received routine coronary angiography, and none of them have acute coronary syndrome.” In addition, all these patients had a cerebral performance category score equal to 1. Unfortunately, we had limited access to the results of CT angiography, especially when patients were referred to our clinic. Following your comment, we have added this into the section of Limitation in lines 412-415 on page 20 as follows: “Patients with a history of aborted SCD in our study had a cerebral performance category score equal to 1. However, we had limited access to the results of CT angiography that might be a routine examination following SCD, especially when patients were referred to our clinic.”

6. Regarding the comment: “In Response 2 to Reviewer 1: “VA and non-VA SCD included” but on row 93-94 ”A shockable rhythm was presented as the initial rhythm or during resuscitation.” What does this mean?”

Thank you for this comment very much. There are separate questions in Response 2, and Reviewer 1 asked that “Were there also patients who presented with non-shockable rhythms initially but who had shockable rhythms later on during resuscitation?” In our study, our record for shockable rhythms(or VA) did not clearly mention whether the shockable rhythms were the initial rhythm or happened during the resuscitation. Therefore, we have to add a statement in the section of Methods to clarify this part in lines 93-94 on page 4 as follows: “a shockable rhythm was presented as the initial rhythm or during resuscitation.”

7. Regarding the comments: “Why PL is not in Table 1? Also some characteristics are rather surprising as the prevalence of smoking, hyperlipidemia, and hypertension are really low, at least compared to Western cohorts. How were the presence of these conditions defined/ascertained? Also, it seem really surprising, that no subjects presented with LVH or BBB in the propensity matched analysis, and the number of subjects with T-wave inversions are really low, although one subject in in the example ECG:s almost fulfills Sokolow criteria, and two subjects present with pathological T inversions. Are these figures correct?”

Thank you for these comments very much. For the first question, we do have the data of PL (Percentage of loop area) in Table 1, and it is at the bottom. In addition, we agree with you that the prevalence of smoking, hyperlipidemia, and hypertension was relatively low, and it was probably related to the selection and survivorship bias that we mentioned in the Section of limitation. Regarding the last part of the comments, it is reasonable that there were no LVH or BBB in the propensity-matched analysis because those patients probably have been excluded. For the last question, we have to say that these figures are correct, and we agreed with you that two of them had pathological T inversions. However, none of them fulfill Sokolow criteria, and we have modified Figure 2 to provide a scale to clarify this.
