# Supplementary material for: Using QRS loop descriptors to characterize the risk of sudden cardiac death in patients with structurally normal hearts
Source: PLoS One. 2022 Feb 16;17(2):e0263894. doi: 10.1371/journal.pone.0263894 (PMC8849494; doi:10.1371/journal.pone.0263894)
Supplement: Attachment — Submitted filename: Response_to_reviewers_R3.docx [file pone.0263894.s008.docx]

We thank the reviewer for the very useful comments. Those comments were very instructive and very helpful to this manuscript. You will find our response to your comments below (in blue color).

Response to Reviewer #2

Thank you for evaluating our manuscript and providing us with valuable comments. Following your comments, we have modified several parts of our manuscript accordingly.

1. Regarding the comment: “The present study compares ventorcardiographic ECG parameters between subjects with aborted SCD and subjects having palpitations due to AVNRT. Subjects with LV hypertrophy, reduced LVEF, regional LV wall motion abnormalities, and congestive heart failure were excluded. The authors use propensity score matching to create equal groups for comparisons according to age, sex, hypertension, and coronary artery disease. The main findings of the study are that mean QRS duration, V4-5 dispersion, and percentage of loop area were different between subjects with aborted SCD and symptomatic AVNRT. These parameters were also associated with SCD independently of the presence of other ECG abnormalities in a multivariate model. Of note, approximately 20% of the SCD cases had V4-5 dispersion >60, whereas none of the controls had V4-5 dispersion >60 and this is an interesting and important finding.”

Thank you for your valuable comments to emphasize the important issues of our study, and we have revised the sentence in the paragraph of main finding accordingly to enforce our points of view, in lines 317-319 on page 16 as follows: “In this study, surface ECG was used to create a vectorcardiogram, which could define the difference between patients with symptomatic AVNRT and patients with a history of aborted SCD.” In addition, we also added the sentence in the same paragraph in lines 322-324 on page 17 as follows: “these parameters were also associated with SCD independently of the presence of other ECG abnormalities in a multivariate model. Of note, approximately 20% of the SCD cases had V_4-5_ dispersion >60**°**, whereas none of the controls had V4-5 dispersion >60**°**.”

2. Regarding the comment: “The application of these results to clinical practice is cumbersome for numerous reasons. The distributions of QRS duration and percentage of loop area were largely overlapping between the cases and controls (despite statistically significant differences in the means), and most of the SCD cases also had V4-5 dispersion in the same range as the controls. Thus, if one tried to screen subjects with structurally normal hearts (generally a really low-risk population) to identify subjects at risk for SCD, only V4-5 dispersion >60 might be of use, and this marker would identify only 20% of cases.

The SCD cases in the present study are not very representative, as none of the cases had acute coronary syndrome (the most common trigger of SCD in the population), and generally no cases with DCM or HCM were included (all patients in the study had normal LVEF and LV wall thickness). However, it would be of great interest if the ECG parameters studied would be predictive of SCD in the aforementioned patient groups.

Therefore, channelopaties were identified in a large proportion of cases (8 Brugada, 6 ARVC, 3 LQTS) and there may have been even more cases as routine cardiac magnetic resonance imaging, or screening for gene mutations associated with these diseases were not routinely performed (at least according to the manuscript). However, as electrocardiograpic characteristics in these diseases (Brs, ARVC, LQTS) differ greatly, the prognostic significance of an electrocardiographic abnormality should be studied separately in each of these diseases.”

We are glad to receive these valuable comments very much. We admit that this study has several drawbacks for widely applying to the general population. As you have mentioned that V4-5 dispersion >60° only identified 20% of cases, but we could also assume that the significant dispersion is probably located at other places. With further analysis of vectorcardiography in subgroups, the subtle abnormalities are likely to be defined and localized.

Regarding the representativeness of our SCD cases, we agreed with you that the etiologies of our patients were not very common in the population. However, our patients referred from other medical centers usually met an unrevealed problem that causes SCD events. These events will probably occur again in the future; therefore, it is still of importance to investigate the possible etiologies underneath these patients and plan further work in electrophysiology.

Seventeen out of 58 patients (29.3%) have channelopathies, and we agreed with you that the electrocardiographic characteristics in these diseases differ greatly. Therefore, we will plan further separate studies to evaluate the prognostic significance of electrocardiographic abnormalities based on distinct conditions.

3. Regarding the comment: “In addition, the methodology used does not provide with an opportunity to assess wheter the electrocardiographic abnormalities detected in patients with aborted SCD were caused by the acute event.”

Thank you for this comment very much. We agree that the methodology does not provide an opportunity to assess whether the electrocardiographic abnormalities detected in patients with aborted SCD were caused by the acute events. We have modified the section of limitations in lines 405-407 on page 20 the clarify this as follows: “the ECG parameters were collected and elaborated after the event; thus, the differences found in our study might also have a chance to be a result of these events.”

4. Regarding the comment: “It should be made clear already in the abstract that the study compares ECGs of patients with aborted SCD and patients having an intervention for AVNRT.”

Thank you for this comment. We have revised the abstract accordingly in lines 30-34 on page 2 as follows: “These patients didn't have structural heart diseases, histories of congestive heart failure, or reduced ejection fraction, and they were classified into SCD (with aborted SCD history and cerebral performance category score of 1) and control groups (with an intervention for atrioventricular node reentrant tachycardia and without SCD history).”

5. Regarding the comment: “It should be mentioned in the limitations section, that no cardiac MRI or genetic data were used in the study.”

Thank you for this comment very much. We have revised the section of Limitation according in lines 417-419 on page 21 as follows: “we had limited access to the results of cardiac magnetic resonance imaging, genetic evaluation, and brain computed tomography angiography that might be routine examinations following SCD, especially when patients were referred to our clinic.”

6. Regarding the comment: “The link to supplemental material led to a Word document including a changes traced version of R1, and no supplemental tables and thus the supplemental tables could not be addressed.”

Thank you for this reminder. It could be something wrong with the link system, but we will upload them again.
